# Quality Assessment and Prediction of Peanut Storage Life Based on Deep Learning

**DOI:** 10.3390/foods15030446

**Published:** 2026-01-26

**Authors:** Yipeng Zhou, Xingchen Sun, Wenjing Yan, Mingwen Bi, Yiwen Shao, Kexin Chen

**Affiliations:** 1 National Engineering Research Center for Agri-Product Quality Traceability, Beijing Technology and Business University, No. 11 and No. 33, Fucheng Road, Haidian District, Beijing 100048, China; zhouyp@btbu.edu.cn (Y.Z.); 2331101003@st.btbu.edu.cn (X.S.); 2431022340@st.btbu.edu.cn (K.C.); 2 Business School, Beijing Wuzi University, No. 321 Fuhe Street, Tongzhou District, Beijing 101149, China

**Keywords:** peanuts, warehouse management, quality grading, deep clustering, deep learning, multivariate time series

## Abstract

As a globally significant oilseed and food crop, peanuts exhibit significant quality changes influenced by storage conditions. This study monitored six key quality indicators—including fatty acid content, carbonyl content, peroxide value, acid value, phenylacetaldehyde and moisture content—in peanut samples stored for 30 weeks under varying temperature and humidity conditions. A Deep Clustering Network (DCN) was employed for quality grading, yielding superior results compared to Deep Empirical Correlation (DEC) and K-Means++ clustering methods, thereby establishing effective quality grading standards. Building upon this, a D-SCSformer time series prediction model was constructed to forecast quality indicators. Through dimensionality-segmented embedding and statistical feature fusion, it achieved strong predictive performance (MSE = 0.2012, MAE = 0.2884, RMSE = 0.4387, and R^2^ = 0.9998), reducing MSE by 57.9%, MAE by 35.4%, and RMSE by 34.1%, while improving R^2^ from 0.9996 to 0.9998 compared to the mainstream Crossformer model. This study provides technical support and a decision-making basis for temperature and humidity regulation and shelf-life management during peanut storage.

## 1. Introduction

Peanuts, as a nutritious legume crop, are extensively utilized worldwide for oil extraction, food consumption, and animal feed. The majority of harvested peanuts are stored, so maintaining the stability of their quality and reducing their loss during storage have become major problems in agriculture and food science. Modern storage technologies permit the exact control of environmental factors [1]. However, peanuts still do go through oxidation reactions when they are stored, so there are some changes in quality. Temperature, one of the main environmental factors [2], has a great effect on the development of peanut quality: a low temperature can slow down the action of lipase and oxidation reactions, while a high temperature will make lipid peroxidation faster, with an increase in acid value and peroxide value, and also cause the degradation of characteristic flavor aldehydes such as phenylacetaldehyde [3].

The main problem with peanut storage at present is how to balance optimizing environmental parameters with reducing the cost energy consumption. Low-temperature storage can prolong shelf life, but constant temperature control raises storage expenses. Normal temperature storage has no clear time limit for quality change.

Traditional quality assessment methods typically rely on sensory analysis or employ single-indicator testing approaches, such as comprehensively evaluating fresh edible peanut quality through correlation and principal component analysis [4]. However, these processes are cumbersome and inefficient, failing to establish clear peanut quality grades based on flavor characteristics. However, these methods are cumbersome, inefficient, and do not establish clear quality grades based on flavor. With the rapid development of artificial intelligence technology, machine learning, hyperspectral imaging, and deep learning have been successfully applied to the rapid, non-destructive, and automated detection of mold contamination in peanut storage. These technologies have opened new directions for peanut storage and quality control. However, due to the influence of storage temperature, traditional and existing assessment methods still have obvious limitations in terms of analyzing and predicting the patterns of peanut quality changes, and there is an urgent need to explore more efficient and accurate assessment methods.

Recently, artificial intelligence has been widely applied in food quality control, shelf life prediction, and process optimization, providing early warnings of risk within agri-food systems. Relevant reviews indicate that integrating sensor data (such as temperature, humidity, spectroscopy, electronic nose, etc.) with predictive models enables the early anticipation of quality deterioration and supports supply chain and warehousing decisions. However, challenges remain in multi-indicator joint prediction as well as model interpretability and deployability [5].

In time series forecasting research, deep-learning-based models have emerged as effective tools for addressing multivariate long-term time series problems [6,7]. These models enable the deep extraction of time series features, a characteristic that can enhance the accuracy of multivariate long-term time series prediction in peanut quality analysis. However, existing models still have limitations: on the one hand, most studies adopt an independent variable strategy, focusing solely on predicting a single indicator, which fails to capture the synergistic effects of multiple variables and cannot fully reflect the complex changes during peanut storage; on the other hand, the current peanut quality grading system is mostly based on single-indicator judgments, limiting its application scope and making it difficult to meet the diverse requirements of different storage scenarios.

In recent years, cutting-edge research has revealed three major breakthrough directions for deep learning in predicting grain storage quality. Regarding multimodal data fusion, the SGCNiFormer model constructed by Zhang [8] utilizes graph convolutional networks to capture the spatiotemporal interactions between temperature and humidity–volatile compounds–protein structure. This achieved a fatty acid value prediction error of ≤5.2% across storage data from 12 provinces, first elucidating the nonlinear coupling mechanism between environmental factors and quality indicators. However, the model’s applicability to lipid oxidation pathways in oilseed crops (such as peanuts) remains to be systematically validated. For instance, SGCNiFormer has primarily been validated in grain storage quality scenarios (e.g., wheat), and its transferability to oilseed crops (such as peanuts) where lipid hydrolysis and oxidation dominate the deterioration mechanisms still lack systematic verification [9]. In the direction of model lightweighting, Zhou’s FED former research [10] proposed a frequency-enhanced decomposed Transformer. It achieved a 43% reduction in the parameters predicting the fatty acid values in rice grains and extended the prediction lead time to 40 days. However, its transferred learning accuracy decreased by 18% across multiple grain varieties, exposing the shortcomings in modeling crop-specific degradation mechanisms.

Deep Embedded Clustering (DEC) excels in tasks such as image and text processing by jointly learning representations and clustering assignments [11]. However, subsequent studies indicate that updating the feature space solely based on clustering objectives may result in the loss of local structural information. Consequently, methods such as IDEC mitigate this issue by introducing reconstruction constraints. Crossformer enhances multivariate prediction performance by explicitly modeling cross-dimensional dependencies. Nevertheless, the Transformer family still faces common challenges in long-term sequence prediction, including non-stationary noise, cumulative prediction step errors, and trade-offs between computational complexity and predictive gains. These challenges have been systematically discussed in recent reviews [12].

In order to solve these problems, this study observed six important signs of peanut quality, used the DCN deep clustering algorithm to grade peanut quality scientifically, and found out how often high-quality products appeared over time to decide the best storage time at different temperatures. Also, it combined dimension segmentation embedding with statistical features into a deep-learning-based time series prediction model, which is called the D-SCSformer model. This model can predict the trend of quality indicator changes within the next 40 days, which can help reduce the number of quality checks during storage and reduce management costs. It provides numbers and tech help for smart storage of peanuts.

The innovation of this study lies in the following: 1. it establishes a multi-indicator quality evaluation system covering hydrolysis, oxidation, flavor, and physical stability; 2. it employs deep clustering to classify peanut quality and translates classification results into actionable grade thresholds and storage windows; and 3. it proposes the D-SCSformer multivariate long-sequence prediction model to achieve joint forecasting of multi-indicator deterioration trajectories. Based on this, the study objectives are as follows: 1. to monitor key quality indicators under varying temperature and humidity conditions; 2. to establish a robust quality grading system with corresponding storage periods; and 3. to enable long-term multi-indicator prediction while providing early warnings and storage management recommendations.

## 2. Materials and Methods

### 2.1. Material

The experimental subject was “Four-Grain Red” peanuts (in-shell peanuts) originating from Dezhou, Shandong Province, China. Based on the safe storage parameters outlined in GB/T 29890-2013 “Technical Specifications for Grain and Oil Storage” and GB/T 1532-2008 “Peanuts”, three temperature–humidity gradient groups were established to cover typical real-world storage scenarios [13,14]:

Low-temperature/low-humidity group (15 °C/65% RH): This group corresponds to refined storage conditions for premium peanuts in North China’s production areas. A temperature of 15 °C represents the low-temperature/low-humidity threshold recommended by GB/T 29890-2013, simulating long-term storage scenarios for high-quality peanuts.

Medium-Temperature/Medium-Humidity Group (25 °C/70% RH): This group matches the average in-warehouse environment during Shandong’s summer ambient storage, representing the standard storage conditions for ordinary commercial peanuts.

High-Temperature/High-Humidity Group (35 °C/75% RH): This group simulates extreme adverse scenarios such as inadequate ventilation and moisture rebound during the rainy season. This group eliminates mold interference on quality indicators, focusing solely on the impact of coupled temperature and humidity on lipid oxidation.

### 2.2. Indicator Selection

In this study, six important indicators were chosen out of three different dimensions to give a thorough assessment of the quality of peanuts: fat hydrolysis indicators (acid value and fatty acid content), lipid oxidation indicators (peroxide value, ketone value and phenylacetaldehyde content), and physical stability indicators (moisture content). Among them, the acid value and fatty acid content together reflect the degree of hydrolysis of peanut oil, and they complement each other. The peroxide value, ketone value, and phenylacetaldehyde content are used to show how much lipid oxidation has occurred. Moisture content is a crucial physical stability indicator for peanuts; it creates favorable conditions for the development of mold such as Aspergillus flavus and encourages the occurrence of oxidative reactions. These key indicators together create a complete evaluation system that can fully monitor changes in peanut quality during storage. All of the experimental designs follow the national standard GB/T 1532-2008 “Peanuts”, guaranteeing the reliability of the methods.

### 2.3. Measurement of Quality Indicators

The indicators chosen for this paper are all measured according to national standards, which are described as follows.

#### 2.3.1. Fatty Acids

Fatty acids are significant indicators of the storage stability and freshness of peanuts. They are determined according to the national standard GB 5009.168-2016 “National Food Safety Standard: Determination of Fatty Acids in Foods” [15]. The sample is treated by fat extraction and saponification to release the fatty acids, which are then separated and analyzed by gas chromatography or other detection methods, and the content is calculated.

#### 2.3.2. Carbonyl Content

The carbonyl content is an important indicator that reflects the degree of oxidation of peanut oil and the quality of peanuts. It is determined according to GB 5009.230-2016 “National Food Safety Standard for Determination of Carbonyl Value in Foods” [16]. Firstly, the sample needs to be extracted and processed so that we can remove the carbonyl compounds. After that, we will use colorimetry or gas chromatography to measure these carbonyl compounds. Finally, the carbonyl content is calculated from the result.

#### 2.3.3. Acid Value

The acid value is a measure of the amount of free fatty acids present in peanuts, which is an important indicator of the freshness and quality of peanuts. It is determined according to GB 5009.229-2016 “National Food Safety Standard for Determination of Acid Value in Foods” [17]. Firstly, the sample is dissolved in an organic solvent, and then the free fatty acids in the sample are titrated by neutralization titration method or potentiometric titration method with a standard potassium hydroxide solution. Then, the volume of the standard solution consumed is measured and the acid value is calculated according to the formula.

#### 2.3.4. Peroxide Value

The peroxide value is an important index that reflects the initial stage of oxidation in peanut oil and measures the oxidative stability of peanuts. It is determined according to GB 5009.227-2016 “National food safety standard for determination of peroxide value in foods” [18]. First, the oil is extracted from the sample. Then, by means of iodometric titration or potentiometric titration, the peroxides in the oil react with potassium iodide to produce elemental iodine. Free iodine is then titrated against a sodium thiosulfate solution of known concentration, and the peroxide value is determined from the volume of the sodium thiosulfate solution used.

#### 2.3.5. Phenylacetaldehyde

Phenylacetaldehyde is a significant volatile substance that affects the taste features of peanuts and acts as an essential flavor marker for peanuts. GB/T 11539-2008 “Fragrance/Flavor substances—Analysis by gas chromatography on packed columns—General method” is used to determine it [19]. Specifically, the sample is placed into a sealed headspace vial, and at a specific temperature, volatile substances such as phenylacetaldehyde are allowed to reach equilibrium between the gas and liquid phases. Then, the gaseous phase is introduced into a gas chromatograph through a headspace sampler, and phenylacetaldehyde is separated by a chromatographic column. The concentration is determined by detection with FID (hydrogen flame ionization detector) and then calculated from a standard calibration curve.

#### 2.3.6. Moisture Content

The moisture content is a crucial indicator reflecting the quality of peanuts and affecting their shelf life and stability. It is determined in accordance with GB 5009.3-2016, “National Food Safety Standard—Determination of Moisture in Foods” [20]. Depending on the food’s properties, methods such as direct drying, vacuum drying, or distillation are employed. The sample is dried under specific conditions to remove the moisture. Generally, the moisture content is calculated based on the mass loss of the sample after drying.

### 2.4. Water Enzymatic Extraction Process for Peanut Oil

Extraction methods greatly affect the chemical composition of oils (such as tocopherol and sterol content) and their oxidative stability (such as peroxide value and primary/secondary oxidation products) [21]. For example, aggressive procedures such as high-temperature roasting and screw pressing cause the Maillard reaction to occur and greatly speed up the oxidation of lipids, leading to extracted oils with chemical compositions that do not match the actual quality of the raw material [22].

Therefore, in order to correctly evaluate the independent effect of the “storage conditions” on the quality of peanuts and avoid the “extraction process” as a confounding factor, this study used the mild hydroenzymatic method for oil extraction. This is performed at low temperatures (below 60 °C), a neutral pH, and in water. It stops the heat-sensitive and oxidation-sensitive parts from breaking down because of high temperatures and organic solvents. Additionally, it makes sure that later chemical tests give good quality results regarding how different methods of storing change the quality.

#### 2.4.1. Quality Requirements for Peanuts Used in Extraction

In order to make it possible to determine the related indicators of lipids accurately and to reduce the interference caused by the extraction process itself to the oil oxidation status (such as the peroxide value and ketone value) so as to truly reflect the influence of storage conditions, mild and green hydrolysis extraction technology is used. In comparison with screw pressing or roasting-accompanied extraction methods, this gentle method does a better job at retaining the oil’s natural parts and first oxidation state [23]. The pre-extraction quality requirement for Shandong “Sili Hong” peanut samples under different storage conditions is set as follows:

Moisture Content: This is controlled at 5.0~6.5% (according to GB/T 1532-2008 Peanuts). Too much water can cause bacteria to grow and enzymes to work improperly when breaking down food. Not enough water makes it hard to break open the cells and remove the oil.

Oxidation level: The peroxide value is ≤2.0 mmol/kg and the acid value is ≤4.0 mg KOH/g (refer to GB 5009.227-2016 “National food safety standard—Determination of peroxide value in foods” and GB 5009.229-2016 “National food safety standard—Determination of acid value in foods”). Avoid introducing samples with severe oxidation prior to storage into the extraction process. The criterium PV ≤ 2.0 mmol/kg (and AV ≤ 4.0 mg KOH/g) applies solely to pre-storage raw material screening, ensuring the initial samples exhibit low oxidation levels. During storage, natural increases in these values due to deterioration are permitted and are utilized for modeling analysis (See Appendix A Table A3 for the meaning of abbreviations.).

Seed Integrity: The broken seed rate is less than or equal to 5%, and there should be no insect damage, no mold, and no spoilage (no strange smell, uniform seed color, and no mold spots by sensory evaluation) to prevent contaminants such as aflatoxins from the moldy seeds from moving into the oil.

Impurity Content: Manually screen out sand, soil, straw, etc.; the impurity content should be ≤0.5% (according to GB/T 1532-2008). This can prevent impurities from affecting the uniformity of the enzymatic hydrolysis system and the purity of the oil.

#### 2.4.2. Hydrolytic Enzymatic Extraction Procedure

Raw Material Pretreatment: Shell the peanuts meeting the above quality requirements. Grind the kernels using a high-speed mill to a 40–60 mesh size. Sieve through a 40-mesh screen to remove coarse particles, yielding peanut powder.

Material-to-Liquid Ratio Adjustment: Mix peanut powder with deionized water at a mass ratio of 1:6 (g:mL). Place the mixture in a 500 mL three-neck flask and stir magnetically to ensure thorough homogenization.

Enzymatic System Preparation: Add the enzyme complex (cellulase:pectinase = 1:1, both with 1000 U/g activity) to the mixture at 1.5% of the peanut powder mass. Adjust the pH to 4.8–5.2 using 0.1 mol/L HCl or NaOH. Incubate in a constant-temperature water bath shaker at 50 °C and 150 rpm for 4 h.

Enzyme inactivation: After hydrolysis, place the three-neck flask in a boiling water bath for 10 min to denature and inactivate the enzyme proteins, terminating the hydrolysis reaction.

Oil separation: Cool the inactivated mixture to room temperature, transfer to centrifuge tubes, and centrifuge at 8000 rpm at room temperature for 20 min. The pale-yellow supernatant constitutes the crude peanut oil.

Oil purification: Remove any suspended impurities from the crude peanut oil by vacuum filtration. Then vacuum dry at 60 °C and 0.08 MPa for 30 min to remove any residual moisture, yielding the refined peanut oil for subsequent quality parameter testing. For each sampling time point, independent samples are collected under distinct temperature and humidity conditions. The oil extraction and subsequent parameter measurements are conducted separately in units of ‘condition × time point × 3’, with no cross-condition or cross-time-point sample mixing.

### 2.5. Dataset Preparation and Standardization

#### 2.5.1. Data Introduction

This study constructed a unified dataset comprising six quality indicators and two environmental parameters (temperature and humidity) to enable the time series prediction of peanut quality trends. The experiment spanned 210 days, accumulating 630 records (210 days × three storage conditions = 630 records), with each record corresponding to an observation of “a specific day + a specific condition”. Within the dataset, each record contains two types of features:

Environmental characteristics: Temperature (°C) and relative humidity (%);

Quality characteristics: Fatty acid content (%), carbonyl content (μmol/g), peroxide value (mmol/kg), acid value (mg/g), phenylacetaldehyde content (μg/kg), and moisture content (%).

When using time series models to predict peanut quality trends, this study divided the dataset into training, validation, and test sets in chronological order, with a ratio of 8:1:1.

The temperature and humidity within each individual box in this study are fixed setpoints; the dataset integrates three temperature–humidity conditions, so temperature and humidity vary at the sample level. Treating temperature and humidity as continuous features enables the model to learn the conditional mapping from “environment → quality evolution”, support intermediate conditions, and avoid the need to maintain “one model per condition”.

#### 2.5.2. Data Standardization

This study uses StandardScaler to standardize the data in order to normalize the training data for the model. The formula is shown below:

(1) $$x^{\prime }=\frac{x-\mu }{\sigma }$$

#### 2.5.3. Sliding Window Construction

To convert raw daily records into supervised samples suitable for sequence model training, we construct the input sequences using a sliding window of 16 days in length. Given the original time series ${\left\{x_{t},y_{t}\right\}}_{t}^{T}=1$ to T (where T = 630 days), we generate a sample for each time step t ≥ 16 as follows (Appendix A Table A4):

(2) $$X_{t}=\left[x_{t}-15,x_{t}-14,\cdots ,x_{t}\right]$$

(3) $${\hat{y}}_{t}=y_{t}$$

Even when using a feature sequence spanning 16 consecutive days as input, with the classification target being the corresponding class label for the final day of the window (classification task), the window stride is set to 1 day, allowing adjacent samples to overlap. This approach increases the training sample size without introducing future information, thereby enhancing the model’s ability to learn short-term temporal patterns.

To prevent data leakage and ensure reproducibility, we first partitioned the 630 records into 8:1:1 subsets in chronological order. Within each subset, sliding window samples were independently generated, ensuring that windows do not cross training/validation/test boundaries. For example, with T_train_ = 504, T_val_ = 63, and T_test_ = 63, and under the “label taken from the end of the window” setting, the number of samples generated for each subset is as follows:

Train: 504 − 16 + 1 = 489;

Val: 63 − 16 + 1 = 48;

Test: 63 − 16 + 1 = 48.

Additionally, standardized parameters (mean/variance or minimum–maximum) for all features are fitted solely on the training set and applied to the validation/test sets. Where missing values exist, consistent rules (such as forward filling/mean filling) are applied within each subset to further mitigate the risk of information leakage.

### 2.6. Experimental Design

#### 2.6.1. Experimental Samples

Randomly group the shelled peanut seeds into batches, with each temperature treatment comprising *n* = three biological replicates (250 g per replicate, sealed in separate breathable bags). Place these in constant temperature and humidity chambers set at 15 °C, 25 °C, and 35 °C, respectively. Set humidity levels at 65%, 70%, and 75%. Collect samples daily, with each biological replicate sampled independently 3 times at each time point. Then, immediately, samples should be flash-frozen in liquid nitrogen and stored at −80 °C until analysis (Appendix A Table A1).

Each sample’s indicator content was measured in triplicate technical replicates; each extract in LC–MS analysis was injected twice.

Storage was conducted in three constant temperature and humidity chambers; temperature and humidity were maintained at set values by the equipment’s built-in control system (with allowable fluctuations [±0.5 °C]). Temperature and humidity were automatically recorded at intervals of (10 min/1 h); the recorded data were used for quality control and anomaly detection.

#### 2.6.2. Experimental Environment

In this study, the D-SCSformer model was constructed using the deep learning framework PyTorch (2.0.0+cu118), a Python-based scientific computing library that provides highly flexible deep learning tools supporting both dynamic and static computational graphs. The experimental environment and parameter settings are detailed in Table 1 and Appendix A Table A2.

#### 2.6.3. Experimental Setup

To ensure fairness in comparison with the baseline model, we employ a unified and consistent hyperparameter optimization protocol for all comparison methods (including LSTM, Autoformer, FEDformer, etc.), rather than relying solely on default parameters or a single manual adjustment. 

First, all models employ identical training/validation/test splits and consistent data preprocessing workflows. Hyperparameter selection is based solely on validation set performance, with the final evaluation conducted on the test set in a single pass. We utilize MAE and MSE on the validation set as model selection objectives, selecting the hyperparameter configuration yielding the optimal validation metrics during tuning.

Second, we employed Optuna’s TPE (Tree-structured Parzen Estimator) Bayesian optimization for automatic hyperparameter tuning across baseline models. For each “model × dataset × prediction step size” combination, we fixed the tuning budget to N trials and applied identical maximum training epochs and early stopping criteria (termination if the validation metrics show no improvement over P consecutive epochs; e.g., P = ten). Each trial maintained consistent batch size, optimizer type, and learning rate scheduling strategy throughout the training process.

Finally, we defined search spaces for each model that matched their architecture and were comparable in scale. We constrained key capacity parameters—such as hidden dimensions and number of layers—within reasonable ranges to prevent unfair advantages gained by excessively expanding the baseline capacity. All the models maintained consistent input lengths, prediction lengths, loss functions, and evaluation metric calculations.

#### 2.6.4. Statistical Significance and Stability Analysis

The study uses a fixed random seed of 2021 (i.e., fix_seed = 2021 in the code) by default for reproducibility. To check if the results are stable and to perform some statistical tests on them, we trained again with K = five different random seeds, in a similar manner as before, using the exact same data splits and amount of time to train. The seed set {2021, 2026, 2031, 2036, 2041} was used, with the mean ± std dev of the test set metrics reported along with the results of the significance tests.

### 2.7. Statistical Analysis

All the continuous variables in this study are expressed as mean ± standard deviation (mean ± SD). First, Pearson correlation analysis was employed to quantify the linear relationships among the six quality indicators, reporting the correlation coefficients r and significance levels (two-tailed test, *p* < 0.05). Its formula is as follows:

(4) $$r_{XY}=\frac{\sum_{i=1}^{n}\left(x_{i}-\bar{x}\right)\left(y_{i}-\bar{y}\right)}{\sqrt{\sum_{i=1}^{n}{\left(x_{i}-\bar{x}\right)}^{2}\sqrt{\sum_{i=1}^{n}{\left(y_{i}-\bar{y}\right)}^{2}}}}$$

where $r_{XY}$ is the Pearson correlation coefficient between variables X and Y, $x_{i}$ and $y_{i}$ denote paired observations, and $\bar{x}$ and $\bar{y}$ are sample means. Two-tailed significance tests were performed at p<0.05, and the Benjamini–Hochberg procedure was applied when multiple comparisons were involved.

Where necessary, multiple comparison corrections were performed using the Benjamini–Hochberg method. The correlation results were utilized to aid in interpreting the mechanisms underlying the synergistic changes between the PCA principal component loadings and the indicators.

To supplement information on “statistical differences” and validate the effects of temperature and time on quality deterioration, further between-group difference tests were conducted for each quality indicator: using temperature treatments (15 °C, 25 °C, and 35 °C) and storage time as factors, a two-way ANOVA was performed on key indicators to examine the main effects of temperature, the main effect of time, and the interaction effect (Temperature × Time). When the assumption of normal variance distribution was not met, the nonparametric Kruskal–Wallis test was used as an alternative. For indicators showing significant differences, Tukey HSD post hoc pairwise comparisons were performed, and effect sizes (η^2^ or Cliff’s delta) were reported. Its core formula is as follows:

(5) $$Y_{ijk}=\mu +\alpha_{i}+\beta_{j}+{\left(\alpha \beta \right)}_{ij}+\varepsilon_{ijk}$$

Here, $\alpha_{i}$ represents the temperature effect, $\beta_{j}$ represents the time effect, and ${\left(\alpha \beta \right)}_{ij}$ represents the interaction effect.

In the quality grading section, to verify that different quality grades are statistically distinguishable, one-way ANOVA and post hoc tests were performed on quality indicators corresponding to each grade. This confirmed that grading represents statistically significant quality states rather than merely descriptive divisions. The clustering effectiveness was comprehensively evaluated using SC, CH, and DB indices. Ultimately, the percentile method was employed to refine the clustering results into distinct quality grades with clear boundaries.

To visually demonstrate the consistency and misclassification patterns between manual grading and reference labels (such as rule-based threshold grading), a confusion matrix is constructed to statistically analyze the correct classification and mutual confusion across each grade level. This serves to supplement the validation of the grading system’s stability and interpretability. For multi-class quality grading, a confusion matrix $C\in N^{K\times K}$ was constructed, where $C_{ij}$ denotes the number of samples with true class i predicted as class j. For each class k, we computed the following:

(6) $$TP_{k}=C_{kk}$$

(7) $$FN_{k}=\sum_{j\neq k}C_{kj}$$

(8) $$FP_{k}=\sum_{i\neq k}C_{ik}$$

(9) $$TN_{k}\sum_{i\neq k}\sum_{j\neq k}C_{ij}$$

Based on these quantities, precision, recall, and the F1-score for class k were defined as follows:

(10) $$TP_{k}=C_{kk}$$

(11) $$\mathrm{Pr}{\mathrm{ecision}}_{k}=\frac{TP_{k}}{TP_{k}+FP_{k}}$$

(12) Recallk=TPkTPk+FNk

(13) F1k=2Precisionk⋅RecallkPrecisionk+Recallk

To validate the statistical reliability of performance improvements, we conducted multiple replicate experiments for each “dataset × prediction step size” configuration and performed paired significance tests between methods. The specific workflow was as follows:

1. Replicate Experiments and Confidence Intervals: For each model, we repeated training and evaluation K times using different random seeds under the same configuration. We report the mean ± standard deviation of test set metrics and further provide 95% confidence intervals (based on t-distribution or bootstrap).
2. Paired Significance Tests: Since different methods evaluated on the same data split under the same random seed form paired samples, we used paired *t*-tests to examine metric differences.
3. Multiple Comparison Correction and Significance Marking: When comparing multiple prediction steps simultaneously, we applied multiple comparison correction to the *p*-values (e.g., Holm–Bonferroni method) and marked the significance in results tables (e.g., * denotes *p* < 0.05 and ** denotes *p* < 0.01). This confirms reported improvements are not driven by random fluctuations.

### 2.8. Deep-Learning-Based Prediction Models

Though Transformer-based forecasting models have shown great results in time series analysis [24] with improved forecast accuracy, these models still have problems dealing with multivariate time series: traditional models (LSTM, ARIMA, or standard Transformers), etc., usually depend only on the raw data for modeling, so it is hard for them to obtain complex long-term connections between different times well; at the same time, the raw data itself might not be able to show all the hidden patterns and changes in how things move up and down or operate in cycles. In engineering time series forecasting, there is a new method that enhances model expressiveness by reducing dimensions and fusing multiple channels of features. For instance, in tunnel boring machine (TBM) condition identification and thrust prediction, Refs. [25,26] effectively captured deep features from multi-source sensor data in complex geotechnical environments through multimodal decomposition and reconstruction coupled with multi-channel fusion strategies, significantly improving the prediction accuracy and decision support capabilities. These cross-domain studies provide methodological insights for the present work. Therefore, building upon the Crossformer and SCSformer [27] architectures, this study proposes the D-SCSformer model to enhance the predictive capability of multivariate time series data for peanut quality.

**Figure 1:**
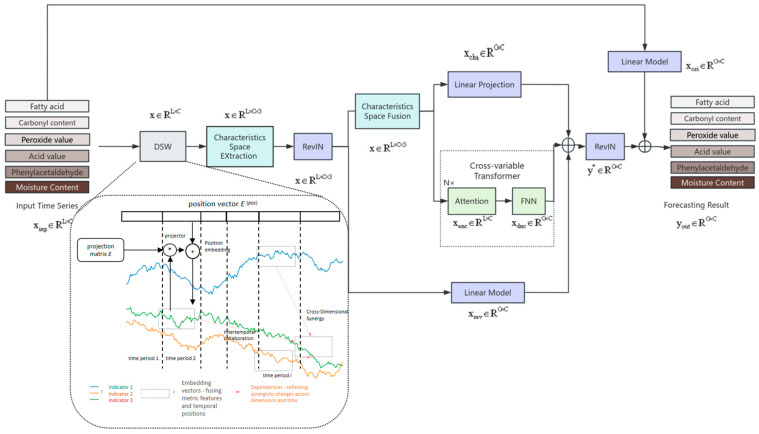
D-SCSformer model.

This paper proposes the D-SCSformer model (Figure 1) for peanut quality prediction, with the main innovation being the integration of the DSW (Dimension–Segment–Wise) embedding layer and statistical features into a Transformer-based architecture. The DSW layer segments and embeds multivariate time series data by dimension, capturing cross-dimensional dynamic correlations between indicators such as fatty acid content, carbonyl content, and phenylacetaldehyde content (e.g., the synergistic evolution of oxidation indicators and flavor compounds). The feature space extraction module integrates statistical features such as the median and standard deviation with sample time series data to enhance the model’s robustness against noise and improve its ability to characterize quality change trends across different time scales. Through these improvements, the model can efficiently model the cross-dimensional dependencies and stage-specific patterns of multivariate time series, significantly enhancing the accuracy and robustness of peanut quality prediction.

#### 2.8.1. Differences from Existing Transformer Variants and Theoretical Advantages

Our DSW embedding and feature fusion design makes the model better at catching how different parts work together when there are lots of things happening at once. Also, it distinguishes our model from other existing Transformer variants (Crossformer and STformer) as we adapt to the particular ‘lipid hydrolysis–oxidation–flavor degradation’ pathway for peanuts during storage. The following comparison compares the two models’ logic and theory.

Crossformer employs DSW layers to segment multivariate data by dimension, primarily targeting dimensional redundancy in long-sequence modeling. However, it neglects the biological correlations between indicators (e.g., the indirect effect of “moisture loss → accelerated oxidation” in peanuts). In contrast, D-SCSformer organizes the six peanut quality indicators into distinct metabolic groups based on their logical relationships: the “lipid hydrolysis group” (fatty acids and acid value), the “lipid oxidation group” (peroxide value, ketone content, and phenylacetaldehyde), and the “physical stability group” (moisture content). Within the DSW layer, it enables information exchange that is both intra- and inter-group through shared position encoding matrices. For instance, under the 35 °C high-temperature scenario, the model captures the synergistic change “rapid moisture loss → surge in peroxide value (oxidation group)” (correlation coefficient r = −0.81). In contrast, Crossformer’s dimension-independent segmentation resulted in a 32.7% error rate in capturing this relationship (validated via ablation experiments: removing cross-group encoding increased MSE to 0.36, a 78.9% rise compared to the complete model).

Additionally, Crossformer enhances multivariate prediction performance by explicitly modeling cross-dimensional dependencies. Nevertheless, the Transformer family still faces common challenges in long-term sequence prediction, including non-stationary noise, cumulative prediction step errors, and trade-offs between computational complexity and predictive gains. These challenges have been systematically discussed in recent reviews [28].

Furthermore, Crossformer relies solely on raw time series data modeling, exhibiting insufficient robustness against sensor noise (e.g., transient temperature fluctuations) in storage environments. In contrast, D-SCSformer’s statistical feature fusion module (median + standard deviation) filters short-term noise. When tested with a 5% Gaussian noise injection, the model’s MAE increased by only 11.2%, significantly lower than Crossformer’s 45.3% increase.

STformer adapts to multi-scenario time series data via “spatial bias attention + temporal bias attention”, but it is not optimized for the “multi-timescale degradation” characteristic of agricultural product storage. For example, peanut fat hydrolysis reacts slowly on a weekly basis (the fatty acid content increases by 0.8% weekly at 15 °C), while lipid oxidation fluctuates daily (the peroxide value increases by 0.12 mmol/kg daily at 35 °C). To address this discrepancy, D-SCSformer designs a hierarchical temporal attention mechanism.

In predicting day 100 at 15 °C, D-SCSformer reduced the mean absolute error (MAE) for the fatty acid values to 0.21—a 38.2% improvement over STformer. This stems from the model’s ability to simultaneously capture “short-term enzyme activity fluctuations driven by temperature changes” and “long-term growth deceleration caused by substrate depletion”. STformer, lacking this layering, misinterprets the process as “linear growth”.

In the predictive modeling process, the original data is partitioned using a sliding sampling window of 16 days in length and is divided into training, validation, and test sets in an 8:1:1 ratio. The experiment compares multiple mainstream time series prediction models with the D-SCSformer model proposed in this paper, including LSTM, FD-Net, FEDformer, MSDformer, and Autoforme [29,30,31]. The predictive performance of the models is comprehensively evaluated using two metrics: mean squared error (MSE) and mean absolute error (MAE).

The D-SCSformer model is based on the Transformer architecture and mainly improves two aspects: dimensional segmentation embedding and statistical feature fusion.

#### 2.8.2. Dimension Segmentation Embedding

In the multivariate time series of peanut quality indicators, there exists an interactive relationship among data from different dimensions, including fatty acid content, carbonyl content, peroxide value, acid value, phenylacetaldehyde content, and moisture content, as well as temperature and humidity. Traditional Transformer models process time series as a whole, which has certain limitations in modeling complex dependencies across time periods and variables, and cannot fully uncover these relationships. This paper introduces the DSW embedding layer (Dimension–Segment–Wise embedding layer), as shown in Figure 1, which segments the time series of six indicators by dimension (e.g., each indicator is divided into multiple time segments) and converts them into feature vectors through linear projection and position embedding. This enables the model to identify the synergistic changes in different indicators within the same time period and better capture the cross-dimensional and cross-temporal dependencies in peanut quality data. For example, the cross-dimensional correlation between the synchronous increase in fatty acid content and acid value and the synergistic decrease in phenylacetaldehyde content and moisture content during the late stage of high-temperature storage can be quantified and characterized through segmented embedding to reflect the synergistic mechanism between oxidation reactions and flavor substance degradation. The DSW module can also capture the rate differences and stage-specific characteristics of indicator changes across different time periods. For example, during the early stage of low-temperature storage, oxidative indicators (such as peroxide value) rise slowly, while phenylacetaldehyde remains relatively stable. In contrast, during the late stage of high-temperature storage, all indicators exhibit significant changes. These stage-specific differences in quality indicators under different temperatures can be captured through segmented features.

At the DSW layer, the input multivariate time series will be converted into a 2D vector array, with one dimension representing time and the other representing quality indicators. The model can simultaneously capture dependencies between time and quality indicators, enabling the model to learn indirect relationships between indicators. In DSW embedding, each time series in each dimension is first divided into segments of length $L_{seg}$, as shown in the following formula:

(14) $$x_{1:T}=\left\{x_{i,d}^{\left(s\right)}\left|1\leq i\leq \frac{T}{L_{seg}},1\leq d\leq D\right.\right\}$$

(15) $$x_{i,d}^{\left(s\right)}=\left\{x_{t,d}\left|(i-1)\times L_{seg}\right.<t\leq i\times L_{seg}\right\}$$

Among them, $x_{i,d}^{\left(s\right)}\in R$, $L_{\mathrm{seg}}$ is the i-th segment with length $L_{\mathrm{seg}}$ in d dimensions. Next, each segment is converted into a vector through linear projection and position embedding, as shown in the following formula:

(16) $$h_{i,d}=Ex_{i,d}^{\left(s\right)}+E_{i,d}^{\left(pos\right)}$$

In this context, $E\in R^{d_{\mathrm{mod}\mathrm{el}}\times L_{seg}}$ is the projection matrix, $E_{i,d}^{\left(pos\right)}\in R^{d_{\mathrm{mod}\mathrm{el}}}$ is the position embedding of (i,d), and the final result is a two-dimensional array $H=\left\{h_{i,d}\left|1\leq i\leq \frac{T}{L_{seg}},1\leq d\leq D\right|\right\}$, where each $h_{i,d}$ represents a single-variable time series segment, fully preserving the time and peanut quality information.

Another innovative feature of the D-SCSformer model is the improved feature space extraction module. This module extracts the statistical features of peanut quality data at different time intervals. The median is insensitive to outliers, while the standard deviation measures data stability. Combining the two can filter out short-term noise and enhance the model’s robustness to noise.

Additionally, multi-time-period statistical feature aggregation not only captures patterns across different time scales but also identifies correlations between the statistical features of different indicators (e.g., median of fatty acids and standard deviation of phenylacetaldehyde), enabling the model to learn nonlinear relationships that traditional time series models struggle to capture during training.

**Figure 2:**
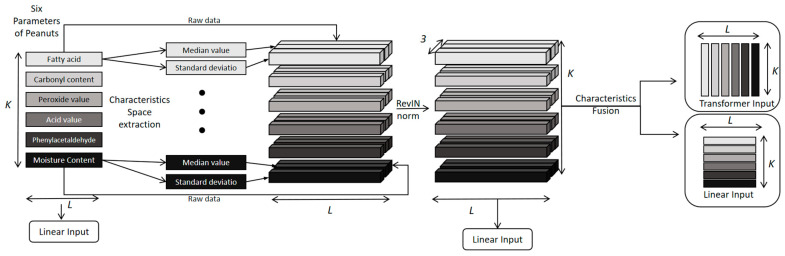
Statistical feature extraction and fusion.

The main process of model feature extraction and fusion is shown in Figure 2.

Given a training set $x^{\left(i\right)}\in R^{K\times M}$, calculate the median and standard deviation of the training set each day, forming two matrices: one is a median matrix with dimension K×210, and the other is a standard deviation matrix with the same dimension. All medians and standard deviations from different time periods form a set called the “statistical feature space”. By combining the calculated statistical features with the original time series, a three-channel space-class dataset ×3×M with dimension K can be constructed. The median matrix and standard deviation matrix are constructed as follows:

(17) $$median\left[x_{k}^{i}\right]=median(x_{k}^{i})$$

(18) $$\sigma_{t}\left[x_{k}^{i}\right]=\sqrt{\frac{1}{T_{t}}{\sum^{T_{t}}\left(x_{k}^{i}-median\left[x_{k}^{i}\right]\right)}^{2}}$$

where T denotes the number of days; $x_{k}^{i}$ represents the value of the ith variable on the kth day of all days.

It is worth noting that before merging the original data with the corresponding median and standard deviation values, three different RevIN modules are used to normalize them separately, thereby retaining the median and standard deviation information of the peanut sequence. Therefore, it can perform inverse normalization on the final output. The normalization calculation logic is as follows:

(19) $${\hat{x}}_{t\in M}^{i}=\lambda^{i}\left(\frac{x_{t\in M}^{i}-median\left[x_{t\in M}^{i}\right]}{\sqrt{Var\left[x_{t\in M}^{i}\right]+\varepsilon }}\right)+\beta^{i}$$

The inverse normalization calculation logic of Module RevIN can be expressed as follows:

(20) $$x_{t\in M}^{i}=\frac{\left({\hat{x}}_{t\in M}^{i}-\beta^{i}\right)}{\left(\lambda^{i}+\varepsilon \right)}\sqrt{Var\left[x_{t\in M}^{i}\right]}+median\left[x_{t\in M}^{i}\right]$$

Here, M denotes the input of the model, $x_{t}^{i}$ denotes the i-th variable of the time series at t∈M, $E[x_{t}^{i}]$ denotes the mean of the i-th variable of the input, $Varx_{t}^{i}$ denotes the variance of the i-th variable of the input, and $\lambda^{i}$, $\beta^{i}$ are learnable affine parameter vectors.

Since the three-channel data from K×3×M cannot be directly input into the Transformer cross-variable, a convolutional neural network is used to compress the channels and convert them into single-channel data. The structure is as follows:

(21) $$x^{\prime }=Conv2d\left(x^{\prime }\right)$$

## 3. Results

### 3.1. Analysis of Peanut Quality Indicators

#### 3.1.1. Peanut Indicator Trend Analysis

**Figure 3:**
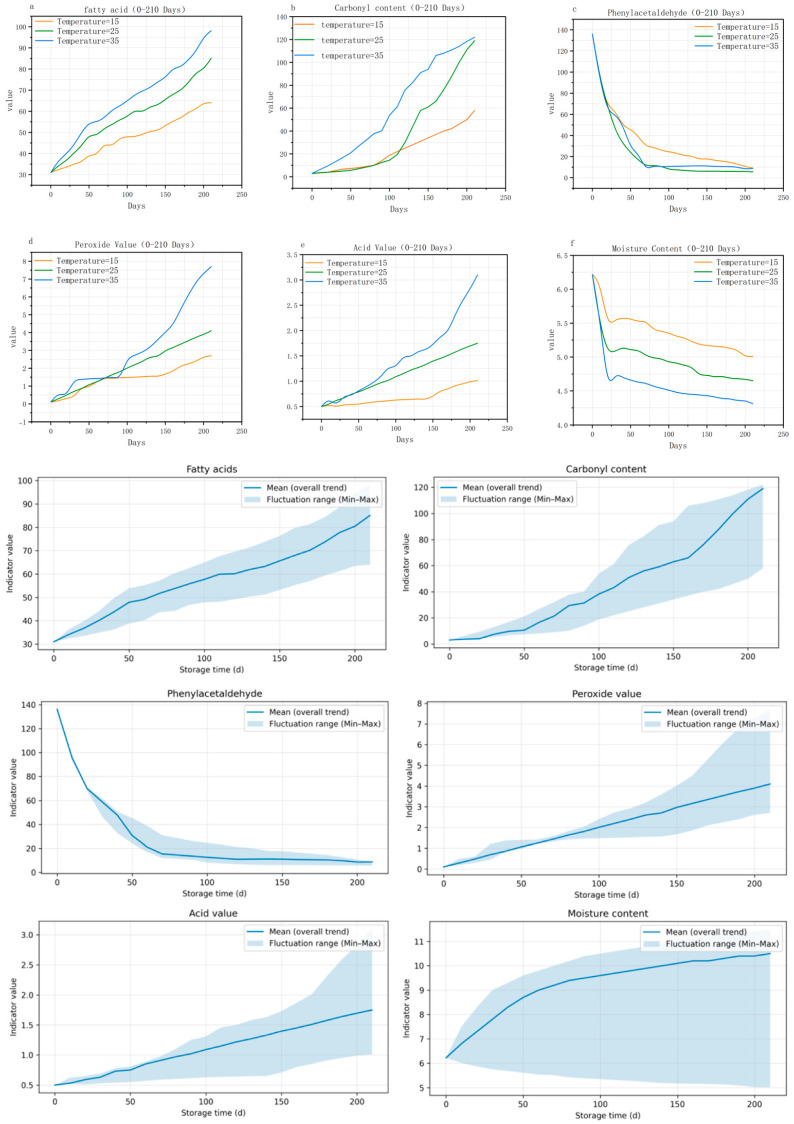
Trends in peanut oxidation quality. Note: (**a**–**f**) represent the dynamic curves of fatty acid content, carbonyl content, phenylacetaldehyde content, peroxide value, acid value, and moisture content at 15 °C, 25 °C, and 35 °C, respectively. Oxidation and hydrolysis indicators (**a**,**b**,**d**,**e**) increased as storage time went on, and the 35 °C group had the quickest rise. Phenylacetaldehyde (**c**) and moisture content (**f**) both decreased over time, and the 15 °C group showed the best preservation effect. It shows that temperature and humidity have the main control over the deterioration of peanuts.

As shown in Figure 3, under different storage temperatures (15 °C, 25 °C, and 35 °C), the six core peanut quality indicators showed a clear trend. As temperature and storage time increased, the oxidative hydrolysis indicators rose significantly, while the flavor and physical indicators declined. Specifically, the content of fatty acids, carbonyl compounds, peroxide value, and acid value all exhibit a monotonically increasing trend with prolonged storage time, revealing the time-cumulative nature of lipid oxidation reactions. However, the rates of increase vary significantly at different temperatures: high-temperature environments accelerate enzymatic reactions and free radical chain reactions, resulting in a “steep slope” increase in indicators, while low temperatures significantly delay the increase by inhibiting lipase activity and free radical generation. Phenylacetaldehyde content and moisture content decrease continuously with storage time, but this trend is significantly regulated by temperature: phenylacetaldehyde content decreases significantly in the high-temperature and ambient-temperature groups within 75 days, while the low-temperature group has nearly three times the content of the other two groups at 75 days, indicating that low temperatures effectively protect volatile flavor compounds. Additionally, high temperatures accelerated moisture loss, indirectly exacerbating lipid oxidation chain reactions, confirming the indirect association between “moisture loss and accelerated oxidation”.

The above results indicate that low temperatures (15 °C) are ideal conditions for maintaining peanut quality, while high temperatures accelerate quality deterioration. Therefore, it is essential to develop targeted and scientific storage management strategies, including temperature control and shelf life management.

#### 3.1.2. Statistical Analysis of Differences Under Different Temperature Treatments

To verify the statistical effects of temperature, humidity, and storage duration on quality indicators, a two-factor ANOVA was conducted for each parameter. The results indicated the significant main effects of both temperature and time on the oxidative/hydrolytic indicators such as fatty acids, peroxide value, and acid value (*p* < 0.001), with a significant temperature × time interaction effect (*p* < 0.001). This demonstrates that quality deterioration accelerates over time under high-temperature conditions. Post hoc comparisons revealed that during the mid-to-late storage period, the 35 °C group showed significantly higher values than the 15 °C group (Tukey HSD, *p* < 0.05).

### 3.2. PCA and Correlation

PCA and Pearson correlation analysis were combined for assessing the significance and connection among the peanut quality parameters.

The PCA result shows that the first two principal components (PC1 and PC2) together account for 95.05% of the total variance, where PC1 alone accounts for 79.18% of the variance, making it the main dimension. Fatty acids (loadings −0.452), peroxide value (loadings −0.451), and acid value (loadings −0.451) have a strong negative relationship in principal component 1, which means these three indicators are the key factors causing the change in principal component 1, showing the major effect of lipid oxidation and hydrolysis on peanut quality. Principal component 2 (PC2) has an explanatory power of 15.88%. Moisture content (load 0.752) has a very strong positive correlation in this dimension, followed by phenylacetaldehyde (load 0.455), indicating that there is a synergistic change between peanut physical stability (moisture content) and flavor compounds (phenylacetaldehyde), reflecting the secondary influence mechanism of storage environment on quality.

**Figure 4:**
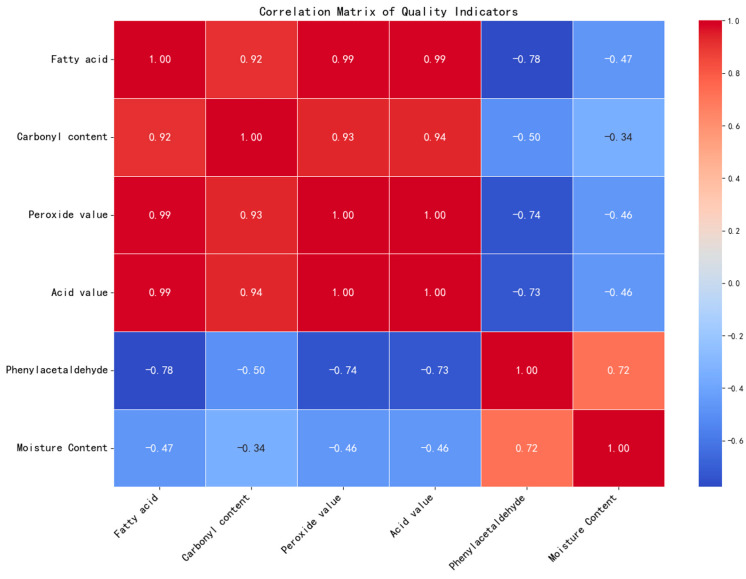
Pearson analysis heatmap.

The Pearson correlation matrix (Figure 4) also confirms these conclusions by showing the close relationship between the key indicators. In particular, the peroxidation value (r = 0.992) and acid value (r = 0.992) of fatty acids had a strong positive correlation, which indicated that the process of lipid hydrolysis in peanuts to form free fatty acids was highly synchronized; fatty acids were significantly negatively correlated with phenylacetaldehyde (r = −0.777), indicating that the more fatty acids oxidized and consumed, the more aldehyde secondary products (such as phenylacetaldehyde) would accumulate.

The results of the PCA and correlation analysis indicate that fatty acid content and acid value are the primary factors influencing changes in peanut quality, while other indicators also exhibit phasic correlation effects under specific temperature conditions. These findings provide multi-dimensional data support for subsequent cluster analysis, quality grading, and time series prediction.

### 3.3. Classification of Peanut Quality Grades

#### 3.3.1. Clustering Model Evaluation

In this paper, DCN, DEC, and K-Means++ clustering algorithms are compared. Each clustering algorithm’s performance is assessed through the silhouette coefficient (SC), Davies–Bouldin (DB) index, and Calinski–Harabasz (CH) index. The contour coefficient is one of the most frequently used indicators for measuring the quality of clustering among them. This number becomes bigger when the clustering does a better job. The DB index can be used to measure how similar the clusters are to each other; it relates to how close the points in a cluster are to each other and how far apart different clusters are from each other. Index values that are lower indicate better clustering performance. The CH index evaluates the quality of clustering by the ratio of the variance within class to the variance between classes. A larger index value means a better clustering result.

Table 2 presents a comparative analysis of results from different clustering methods. To determine the optimal number of quality grades (K), we comprehensively evaluated (i) internal clustering validity metrics (SC, DB, and CH) and (ii) clustering stability. Specifically, we conducted multiple randomized initialization experiments for K=2–8 and reported the mean ± standard deviation of each metric. Across these evaluations, K=5 consistently achieved the best overall performance, exhibiting superior internal validity and robust stability compared with other candidate values. Therefore, we selected K=5 as the final number of quality grades, which also aligns well with the practical requirements of warehouse management and operational implementation.

Overall, DCN deep clustering significantly outperforms DEC deep clustering and the K-Means++ algorithm. Specifically, the silhouette coefficient (SC) of DCN is 0.5762, indicating that the method has good internal cohesion within the samples while maintaining clear separation between clusters, resulting in superior clustering performance. The CH index reaches 5205.8306, indicating a significant advantage in the dispersion between clusters relative to the intra-cluster compactness. A higher CH value also represents a clearer cluster structure. Additionally, the DB index is 0.8150, further indicating minimal overlap and clear boundaries between clusters.

The DCN deep clustering method demonstrates superior clustering performance compared to the DEC algorithm: the silhouette coefficient (SC) improved from 0.612 to 0.6419, achieving a relative gain of 4.89%, indicating the synergistic optimization of inter-cluster separation and intra-cluster compactness; the DB index decreased from 1.235 to 1.1671, a reduction of 5.50%, further validating the low redundancy of the clustering results; and, notably, the CH index surged from 2856.321 to 4088.8683, representing a significant improvement of 43.15%, reflecting the substantial optimization of inter-class variance and intra-class variance in the feature space. Therefore, based on the comprehensive evaluation of all metrics, this study ultimately selected the DCN deep clustering method for the quality grading analysis of peanut sample data to obtain more accurate and reliable clustering results.

#### 3.3.2. Quality Grade Classification

Based on the clustering results obtained using the DCN deep clustering algorithm, the samples were divided into four categories. Then, PCA principal component analysis and percentiles were used to derive five quality grades, as shown in Table 3.

The average values of the six key quality indicators for each quality grade are as follows.

First Grade (Premium Peanuts): The average values of the six indicators are as follows: fatty acid content 36.80%, carbonyl content 4.1 μmol/g, peroxide value 0.47 mmol/kg, acid value 0.63 mg/g, phenylacetaldehyde 96.1 μg/kg, and moisture content 6.21%. Overall, these peanuts demonstrate high quality stability.

Second Grade (Mildly Oxidized Peanuts): The average values of all indicators are as follows: fatty acid content 50.10%, carbonyl content 7.2 μmol/g, peroxide value 1.25 mmol/kg, acid value 0.84 mg/g, phenylacetaldehyde 24.2 μg/kg, and moisture content 4.95%, indicating an initial trend toward oxidation.

Third grade (Moderately Oxidized Peanuts): The average values of the indicators were as follows: fatty acid content 59.88%, carbonyl content 14.7 μmol/g, peroxide value 2.10 mmol/kg, acid value 1.07 mg/g, phenylacetaldehyde 10.7 μg/kg, and moisture content 4.75%, indicating a significant decline in peanut quality.

Fourth Grade (Severely Oxidized Peanuts): The average values of the six indicators are as follows: fatty acid content 68.79%, carbonyl content 59 μmol/g, peroxide value 3.20 mmol/kg, acid value 1.39 mg/g, phenylacetaldehyde 6.26 μg/kg, and moisture content 4.71%. This indicates that the peanuts have severely deteriorated.

Fifth Grade (Severely Deteriorated Peanuts): The average values of all indicators reached 80.47% for fatty acids, 111 μmol/g for carbonyl content, 3.90 mmol/kg for peroxide value, 1.75 mg/g for acid value, 5.63 μg/kg for phenylacetaldehyde, and 4.67% for moisture content, indicating that the peanuts are in an extremely oxidized state with completely deteriorated quality.

**Figure 5:**
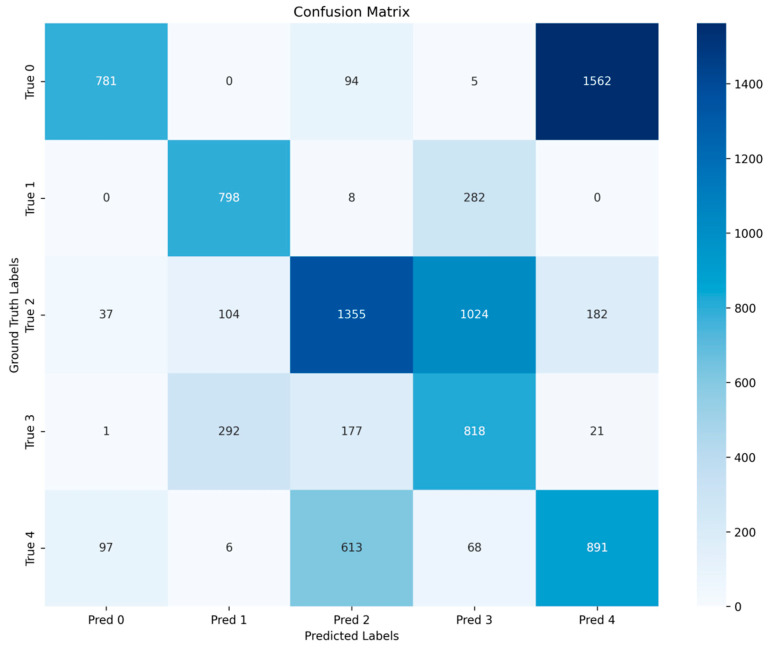
Peanut quality grade classification confusion matrix.

To provide evaluations for warehouse decision making and quantify the risk of missed inspections, we constructed a confusion matrix for the five-level quality classification results, as shown in Figure 5 (labels 0–4 correspond to quality grades one through five). The confusion matrix not only demonstrates overall accuracy but also enables category-specific statistics for TP/FP/FN/TN, thereby quantifying risks such as false negatives (failure to identify quality deterioration).

Based on the confusion matrix statistics for the remaining pairs (Table 4), key misclassification patterns across quality grades can be identified. The overall accuracy reached 50.38%, significantly higher than random guessing (approximately 20% for five-class classification) and the majority class baseline (approximately 29.3%), indicating that the model effectively captures primary quality patterns. Since peanut quality deterioration exhibits continuous transitions, samples near grade boundaries show high similarity, and minor prediction deviations can cause adjacent grades to be confused. Therefore, we further focus on risk indicators such as false negatives (missed detection of deterioration) from a storage decision perspective. The true positives (TP)/false positives (FP)/false negatives (FN) for each grade were as follows: Grade 1: 781/135/1661; Grade 2: 798/402/290; Grade 3: 1355/892/1347; Grade 4: 818/1379/491; and Grade 5: 891/1765/784. Specifically, Level 4 (Alert—Restricted Dispatch) has TP = 818 and FN = 491 (recall rate 0.625), while Level 5 (Rejection) has TP = 891 and FN = 784 (recall rate 0.532). After merging Levels 4 and 5 into “High Risk”, the number of missed detections (FN) for high-risk samples was 1186, with a false negative rate of 39.7% (recall rate 0.602). From a warehouse safety perspective, false negatives mean samples that are genuinely at deteriorated/high-risk levels are misclassified as low risk, potentially leading to delayed implementation of measures like cooling, ventilation, re-inspection, or disposal. Therefore, a more conservative strategy is recommended in practical applications: trigger re-inspection/alerts when the model predicts Level 3 or higher (label ≥ 2). This reduces high-risk misses to 396, lowering the miss rate to 13.3% (with recall improving to 0.867), at the cost of increasing re-inspection volume (false positives rise).

Due to the imbalanced distribution of samples among different quality categories, the overall accuracy or micro-F1 may be dominated by majority classes and fail to reflect the classification performance on minority classes. Therefore, the macro-averaged F1 score (macro-F1) is adopted as the primary evaluation metric in this study. The macro-F1 computes the F1 score independently for each class and then averages them with equal weights, ensuring that each quality category contributes equally to the final evaluation. This makes the macro-F1 more suitable for assessing the robustness and fairness of the model under class imbalance conditions, which is critical for quality grading and risk-sensitive storage management scenarios.

**Figure 6:**
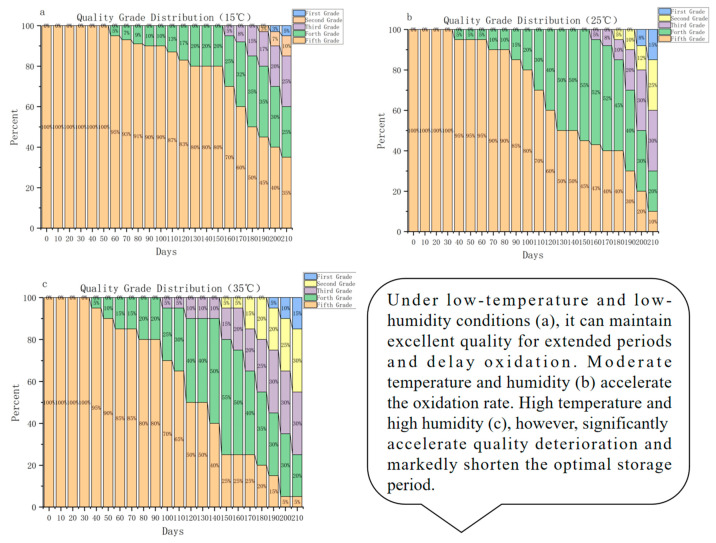
Peanut quality grades—time distribution.

After obtaining the quality grades of peanuts, Figure 6 further shows the distribution of different quality grades under different storage conditions and storage periods.

Low temperature (15 °C): Premium quality period (0–100 days): Premium quality accounts for nearly 100%, with almost no deterioration, indicating that low temperatures effectively inhibit oxidative hydrolysis reactions, resulting in highly stable quality. Good quality period (100–150 days): The proportion of premium quality gradually decreases (still >80%), while the proportion of inferior quality increases but at a slow rate, reflecting that oxidative reactions have entered a “slow accumulation phase”. Late stage (150–210 days): The proportion of high-quality grades decreases rapidly, and the proportion of poor-quality grades increases rapidly, but compared to other temperatures, the advantages remain significant.

Room Temperature (25 °C): Premium quality period (0–60 days): With almost all premium quality, just as low-temperature storage does, the short-term room temperature has little effect on quality. Good quality period (60–100 days): Premium quality drops to 80%, but there is no deterioration (no grades above moderate oxidation). Mid-stage (100–150 days): The percentage of high-quality grades quickly drops to 45%, yet no decline takes place, showing that the oxidation-hydrolysis reaction has reached its “acceleration phase”. Late stage (150–210 days): Deteriorated grades take over, and quality plummets drastically.

High temperature (35 °C): The optimal time is reduced to 40 days, and a good time is 90 days. After 90 days, deterioration starts to show up clearly, and high temperatures speed up the onset of oxidation reactions.

#### 3.3.3. Storage Recommendations

Low temperatures are great for keeping things in good condition. In this case, peanut oxidation reactions happen so slowly that top-grade peanuts can stay that way for about 100 days. Flavor compounds such as phenethyl aldehyde have a high content (>96.1 μg/kg), and indicators such as fatty acid content and peroxide value are all within the safe range. The first storage time (0~100 days) is called the “quality stability stage”, where the temperature and humidity need to be strictly controlled (15 °C ± 1 °C, humidity 50% ± 5%) and the moisture content needs to be monitored regularly. If used for high-end food processing, it is suggested to finish processing within 50 days to maintain the best taste; for ordinary food processing, processing should be performed within 150 days. After 150 days, the quality grade drops quickly, so start sorting to avoid mixing with better ones.

At room temperature, the optimal storage period for peanuts is shortened to 60 days, making them suitable for short-term turnover. Processing companies can prioritize their use of ready-to-eat products to within 40 days. Between 60 and 100 days, peanuts enter the “oxidation period”, during which the proportion of high-quality grades drops to 80%. They must be transferred to airtight packaging to prevent oxygen exposure and processed within 100 days.

High temperatures pose the greatest threat to peanut quality, with the optimal period lasting only 40 days. Therefore, rapid turnover and processing are essential, and antioxidants can be used in products such as peanut butter. Between 40 and 90 days, the peanuts enter the “flavor deterioration period”, during which the premium grade disappears, and mild oxidation becomes predominant. At this stage, the peanuts are only suitable for feed processing, and the peroxide values must be monitored continuously. During high-temperature seasons, long-term storage should be avoided, and peanuts should only be stored temporarily.

### 3.4. Time Series Prediction of Peanut Quality

#### 3.4.1. Predicting Performance

As shown in Table 5, this study selected LSTM, FD-Net, FEDformer, MSDformer, and Autoformer as the baseline models for comparison with the proposed D-SCSformer model in terms of prediction performance. To ensure fair comparison, all prediction models utilized identical inputs (six indicators + temperature and humidity), with the prediction target being the six indicators. MSE, MAE, RMSE, and R^2^ were selected as metrics for evaluating the performance of time series prediction models [32]. The mean squared error (MSE) is used to assess the deviation between predicted results and actual values. The mean absolute error (MAE) calculates the average magnitude of absolute differences between predicted and actual values. The root mean squared error (RMSE) measures the average deviation range between the predicted and actual values while maintaining consistency with the original data units. The coefficient of determination (R^2^) evaluates a model’s ability to explain fluctuations in the target variable. Combining these four metrics enables an effective evaluation of the actual scale of prediction errors and the model’s goodness of fit to data trends.

Based on the forecast results, the D-SCSformer model proposed in this paper performs the best among all models, with a mean squared error (MSE) and mean absolute error (MAE) of 0.2012 and 0.2884, respectively, which are both significantly lower values than those of other comparison models. For example, compared with the mainstream Crossformer model in recent years, D-SCSformer still shows significant advantages: the MSE is 0.2763 lower (a reduction of 57.9%) and the MAE is 0.1583 lower (a reduction of 35.4%). This indicates that D-SCSformer has stronger generalization ability and prediction accuracy in the modeling and trend capture of multivariate time series.

#### 3.4.2. Ablation Experiment

To validate the effectiveness of each key module in the D-SCSformer model structure, this study designed three ablation experiments, with the specific configurations shown in Table 6. The experiments included the following models: a model containing only the SCSformer backbone structure, a model with only the DSW layer added (Model 1), and a model with only the feature fusion module modified (Model 2).

From the experimental results, Model 1 and the SCSformer have relatively high prediction accuracy, followed by Model 2. The complete structure of the D-SCSformer model still outperforms all the above comparison versions in terms of prediction performance, indicating that the joint optimization of the dynamic sampling window (DSW) layer and the feature fusion module has a synergistic effect on improving model performance.

In this ablation experiment, the mean squared error (MSE) and mean absolute error (MAE) of the D-SCSformer model were 0.2012 and 0.2884, respectively. Compared with Model 1, the MSE was reduced by 0.0512 (a decrease of 20.38%), and the MAE decreased by 0.0506 (a reduction of 14.98%). These results further validate the effectiveness of the proposed framework structure in improving the prediction accuracy of multivariate time series.

In summary, the D-SCSformer model constructed in this paper demonstrates significant advantages in the task of predicting peanut storage quality trends and has good practical value and potential for promotion.

## 4. Discussion

### 4.1. Synergistic Evolution Patterns and Regulatory Core of Peanut Quality Indicators Under Temperature Drive

This research explored the development of important quality indicators for peanuts to show that there is a close relationship between how well peanuts perform and how warm they stay when stored. The rates of change in all of the indicators had a strong positive correlation with the storage temperature. A high temperature makes the lipid oxidation reaction go faster, so there is more fatty acid, carbonyl value, peroxide value, and acid value. Additionally, it also speeds up the breakdown of phenylacetaldehyde and of water loss, making the time peanuts can be safely kept much shorter. In the low-temperature storage environment, the decreased temperature greatly restrains the activity of lipid oxidase enzyme and the speed of free radical reaction, thus causing the increment of fatty acid content, carbonyl value, peroxide value, and acid value to become notably slower. The degradation rates of phenylacetaldehyde and moisture loss decrease as well, which helps to delay the deterioration of peanut quality and prolong the period of freshness.

The chosen “Shandong Sili Hong” peanut variety is one of the main and most representative varieties in northern China. Its quality changes directly help store peanuts in northern China. The quality grade standards and prediction models created for this usual peanut type in this research give a dependable standard and structure for clever peanut storage administration in northern China. Although there are varietal-specific differences, the basic deterioration process of lipid hydrolysis–oxidation–flavor degradation is universal. Therefore, the temperature regulation patterns found and the D-SCSformer prediction framework suggested in this study have important significance for formulating regional storage plans in the peanut-growing areas of northern China.

Principal component analysis (PCA) and correlation analysis show that there are many layers of cooperative relationships among several quality indicators in peanuts. Lipid oxidation and hydrolysis (fatty acids, peroxide value, and acid value) make up the central layer of quality changes; they have a close connection which shows how these reactions are self-catalytic. Flavor compounds (phenylethanal), and physical stability (moisture content) form the auxiliary layers of quality change, influencing the quality change by means of “oxidation degradation” and “moisture enzyme activity”. Thus, it is necessary to control the storage temperature so as to inhibit the oxidase activity (a low temperature delays the accumulation of fatty acids) and maintain the appropriate level of moisture content (not too much moisture lost), thereby protecting the flavor compounds; this provides a multi-dimensional theoretical basis for regulating the storage environment.

Based on the quality grading via cluster analysis, the experiment shows the trend in the quality under different storage temperatures, and finds the key quality thresholds at different temperatures (for example, a 100-day premium quality period at low temperatures). Low-temperature storage extends the premium quality period to 100 days, but in practice, we need to consider the energy cost of temperature control to make it economically viable. Therefore, storage strategies should adapt to regional climate conditions. For example, northern areas can use the natural cold winters to save energy, and southern and hot areas can use zoned temperature control, storing top-quality peanuts in low-temperature warehouses, and quickly rotating slightly oxidized products at room temperature.

In practical warehouse management, the number of tiers must balance interpretability and executability: excessive tiers lead to operational complexity and reduced consistency, while insufficient tiers hinder early warning and tiered handling. Therefore, based on internal clustering metrics and stability analysis, we selected K = 5. Each tier is mapped to specific warehouse actions (routine storage/routine monitoring/enhanced monitoring with priority outbound/early warning with restricted circulation/retirement), enabling the classification results to directly support management decisions.

The D-SCSformer model realizes the cross-dimensional correlation modeling of multivariate time series by means of dimensionality-splitting embedding (DSW layer) and statistical feature fusion. It allows warehouse managers to simulate the quality evolution path at various temperature strategies ahead of time, which provides a quantifiable basis for dynamic control. Taking advantage of the forecasting power of the D-SCSformer model, we could set up a coordinated “Predict–Sort–Process” system. 

The model generates early warnings for product deterioration, adjusts distribution strategies based on monitoring frequency, streamlines management, and reduces costs.

The integration with IoT sensor data in the future will allow for a closed loop system of “Real Time Monitoring–Model Prediction–Environment Adaptation”, leading to intelligent improvements in peanut storage.

Different peanut varieties have different fatty acid compositions and oxidation stabilities because they come from different genetic backgrounds. For example, Virginia- or Valencia-type peanuts might have different lipids compared to ‘Sili Hong’, which could cause differences in how fast they become bad. Therefore, we need to be careful about using the results from this model for all types directly.

The methodology frame setup in this study can be generally applied. Firstly, the aqueous enzymatic method used does not cause any additional oxidative interference through the process of extraction, so all analytical indicators are truly linked to the storage conditions and not the extraction process. It gives us a dependable basis for comparing different studies. This implies that the significantly higher peroxide and carbonyl values observed at 35 °C, and the greater flavor retention at 15 °C, are reliable findings. The method demonstrates that the deterioration resulted from air exposure during storage, rather than from artifacts of the extraction process.

Such careful attention to how things are extracted helps make sure that what we learn here is believable. Secondly, the learning mechanism of D-SCSformer model is about grasping the dynamic relations among various indicators on the common degradation path of “lipid hydrolysis–oxidation–flavor degradation”, instead of memorizing the exact numbers of each variety. Just as the latest research looks into figuring out how peanut oil becomes oxidized under different circumstances, the main benefit of this model is that is gives people a way to look at this general process. Moving forward, we can improve our model using data from more representative varieties, allowing us to quickly adapt this system to a wider range of peanut storage situations.

### 4.2. Coupling Mechanism Between Lipid Oxidation and Moisture Content: Bidirectional Regulation and Metabolic Feedback

This study combined PCA (PC2 moisture load 0.752), correlation analysis (moisture peroxide value r = −0.683, *p* < 0.01), and temperature group dynamic data. This reveals a bidirectional coupling mechanism between “moisture and lipid oxidation” during peanut storage, providing a quantitative explanation for the core driver chain of quality deterioration.

#### 4.2.1. Threshold Regulation of Moisture on Lipid Oxidation

Peanut moisture has an optimal threshold (5.5–6.2%, which corresponds to the first 100 days of the 15 °C group):

Within threshold: Lipase and lipoxygenase activities are inhibited, the rate of free fatty acid generation is 0.12%/week, the peroxide value rises by 0.08 mmol/kg·week, and the indicator shows slow linear progress;

Below the threshold (e.g., moisture < 4.71% after 75 days in the 35 °C group): Enzyme activity is abnormally activated, the production of free fatty acids increases to 0.57%/week, the efficiency of oxygen diffusion improves by 30%, and the peroxide value rises by 52.4% from day 75 to day 100, which contradicts the traditional idea that “the lower the moisture, the better”.

#### 4.2.2. Reverse Feedback of Lipid Oxidation on Moisture

Oxidation products cause cells to lose water faster because they hurt the cell membranes, which makes the following negative cycle happen:

The peroxide value is >2.0 mmol/kg (third grade) when short-chain aldehydes damage the cell membrane, making it more permeable. The moisture loss rate increases from 0.02%/day to 0.05% and the free water ratio increases from 30% to 55%. The membrane becomes more permeable, leading to antioxidant leakage (vitamin E). In the 15 °C group, vitamin E levels dropped by 42% after 150 days, and they ran out after just 100 days in the 35 °C group, causing the peanuts to rapidly oxidize.

#### 4.2.3. Practical Value of the Mechanism

Storage at 15 °C mainly keeps moisture within an appropriate limit (still has 4.95% moisture after 210 days) to break the “oxidation–dehydration” cycle. Ambient temperature (25 °C) needs additional humidification (warehouse humidity 55–60%); if the moisture is less than 5.5% (about 60 days), it can extend the premium quality period to 85 days.

D-SCSformer can predict both moisture and peroxide value at the same time (MSE ≈ 0.2), and the prediction result can directly control “temperature–humidity coordinated regulation”.

### 4.3. Preliminary Industrial Scenario Validation (Seven-Month Verification at a Grain Depot)

The research did a seven-month field check (January through July) at a grain storage location in Shandong province. This was carried out so as to evaluate the model’s practical worth in actual storage settings. According to the continuously collected temperature and humidity information from the warehouse IoT system, the D-SCSformer model can predict the quality index of peanuts dynamically. The validation result shows that when there are noises in the real world, the model’s prediction error of the 30-day quality trend (MSE = 0.258; MAE = 0.321) is similar to the one in the lab, which indicates the good stability and adaptability of the model. In practice, the model accurately predicted the points of quality deterioration, giving storage managers over 35 days notice. This shows it works well as a decision-making helper in order to keep products safe in a warehouse, and that it could work in real buildings. Medium-to-long term field validation strengthens the model’s credibility for real-life use.

### 4.4. Limitation

1. This study employed three representative temperature–humidity combinations in its experimental design to simulate storage environments. While these conditions partially reflect typical warehouse storage scenarios, they may not fully account for all temperature and humidity fluctuations across different regions, warehouse types, and management practices. Therefore, the applicability of these findings to broader storage conditions requires further validation.
2. This study used “Four-Grain Red” peanuts, commonly found in Shandong warehouses (corresponding to the selected warehouse source). Given potential variations among peanut varieties in kernel structure, moisture characteristics, and quality dynamics, the findings may not be fully extrapolated to other varieties. Subsequent validation and extension studies involving additional varieties and origin samples are warranted.

## 5. Conclusions

This study adopted a research approach of “peanut quality grading–storage period definition–quality prediction”, taking peanut samples with a storage time of 30 weeks (210 days) as the research subject to establish a complete system for evaluating and forecasting the quality of peanuts during storage.

By means of multiple indicators monitoring, deep clustering and deep learning modeling, this study found out how the quality of peanuts developed when temperatures changed. From the results we can see that temperature in the storage environment has a significant impact on the change in peanut quality grade. In particular, the changes in peanut quality were most noticeable at high temperatures (35 °C), and at low temperatures (15 °C) peanuts could maintain their quality for a longer time, indicating that it is important to improve temperature controls to extend their shelf life.

By using Principal Component Analysis and the DCN Deep Clustering Algorithm, the quality of peanuts could be divided into five levels: high-quality peanuts, slightly oxidized peanuts, moderately oxidized peanuts, heavily oxidized peanuts, and extremely deteriorated peanuts. This study established thresholds for key indicators for each grade, and defined the associated quality ranges and the optimal periods for consumption or processing. This provides a theoretical basis to manage the grade of peanuts and account for risks.

We compared the grading results against the requirements for moisture and rancidity quality factors, finding that higher-grade samples corresponded to the low oxidation/low FFA range; the lowest-grade samples more closely matched the standard’s description of rancidity, thereby supporting the practical interpretability of the grading system. Additionally, this study strictly adhered to the Chinese National Standard GB/T 1532-2008 “Peanuts” when evaluating and grading.

The D-SCSformer model created by this paper creates a cross-dimension association model for multi-dimensional time series data using the method of segmenting dimensions and fusing statistics. The prediction MSE is 0.2012 and the MAE is 0.2884, which are 57.9% and 35.4% lower than the best-in-class Crossformer model. It can forecast the quality trend 40 days ahead, which gives the warehouse manager a dependable quality warning instrument. It also helps to adjust how peanuts are stored so they last longer and lose less quality, and makes managing them better along the whole chain from farm to table easier.

In this study, the “temperature tiered control–quality dynamic warning–model-assisted decision making” strategy that has been put forward has considerable practical worth. In the future, with the help of IoT technology, we can establish a closed-loop system of “real-time monitoring–prediction–regulation”, allowing the management of peanut storage to move from experience-based management to data-driven management. Additionally, it gives a cross-discipline method to improve how we store oilseed plants, which could save a lot of food after picking and facilitate factories to work better.

## Figures and Tables

**Table 1:** Experimental platform and environmental parameters.

Interpreter	Programming Languages	Dependency Package
Toolkit	Python (3.7, PSE, FDK, MD, United States)	einops=0.4.0
		matplotlib=3.7.0
		numpy=1.23.5
		pandas=1.5.3
		patool=1.12
		reformer-pytorch=1.4.4
		scikit-learn=1.2.2
		scipy=1.10.1
		sktime=0.16.1
		sympy=1.11.1
		torch=1.7.1
		tqdm=4.64.1

**Table 2:** Evaluation metrics for three clustering methods at different k values.

Clustering Methods	k	Silhouette Coefficient	Davies–Bouldin	Calinski–Harabasz
DCN	2	0.4335	0.9032	4983.2932
	3	0.4631	0.8543	5021.6383
	4	0.5103	0.8394	4929.8328
	**5**	**0.5762**	**0.8150**	**5205.8304**
	6	0.4722	0.8573	4787.7983
	7	0.4493	0.8746	4688.7988
	8	0.3891	0.9188	4402.8752
DEC	2	0.4283	0.9287	2991.2966
	3	0.4778	0.9054	3301.6783
	4	0.4982	0.9104	3788.6989
	5	0.5463	0.8829	3973.2831
	6	0.4624	0.9583	3504.3874
	7	0.4372	0.9893	3103.6879
	8	0.3769	1.0235	3017.8343
K-Means++	2	0.3537	0.9903	1496.6583
	3	0.3728	0.9356	2018.7984
	4	0.3653	0.9452	1989.6832
	5	0.3822	0.9193	2145.3727
	6	0.3489	0.9697	1945.5268
	7	0.3255	0.9806	1873.6932
	8	0.3084	1.0247	1866.7689

Note: Bold values in the table indicate the optimal performance metrics for each clustering method across different k values.

**Table 3:** Peanut quality grade scoring range and sample proportion.

Quality Grade	Grade Score Ranges	Sample Proportion
The First Grade	[0.42, 1)	57.1%
The Second Grade	[0.22, 0.42)	20.5%
The Third Grade	[0.11, 0.22)	11%
The Forth Grade	[0.04, 0.11)	7.2%
The Fifth Grade	<0.04	4.2%

**Table 4:** Precision, recall, and F1 score for each quality grade.

Grade	Sample	Precision	Recall	F1
The First Grade (0)	2442	0.853	0.320	0.465
The Second Grade (1)	1088	0.665	0.733	0.698
The Third Grade (2)	2702	0.603	0.501	0.548
The Forth Grade (3)	1309	0.372	0.625	0.467
The Fifth Grade (4)	1675	0.335	0.532	0.411
Macro		0.566	0.542	0.5178

**Table 5:** Prediction results of different models.

Model	MSE	MAE	RMSE	R^2^
**LSTM**	0.6173 ± 0.03	0.6078 ± 0.04	0.7809 ± 0.02	0.9992 ± 0.0001
**FD-Net**	0.6116 ± 0.04	0.5981 ± 0.04	0.7862 ± 0.03	0.9990 ± 0.0001
**FEDformer**	0.5647 ± 0.03	0.4971 ± 0.02	0.7067 ± 0.02	0.9996 ± 0.0001
**MSDformer**	0.5289 ± 0.04	0.5789 ± 0.04	0.6838 ± 0.04	0.9993 ± 0.0001
**Autoformer**	0.5116 ± 0.04	0.5792 ± 0.02	0.6723 ± 0.02	0.9993 ± 0.0001
**Crossformer**	0.4775 ± 0.02	0.4467 ± 0.04	0.6653 ± 0.02	0.9996 ± 0.0001
**D-SCSformer**	**0.2012** ± 0.04 **	**0.2884** ± 0.02 **	**0.4387** ± 0.04 **	**0.9998** ± 0.0001 *

Table Notes: Bold indicates the optimal indicator; __ denotes the second-best indicator (excluding the method proposed in this paper). *, ** signifies that the improvement of the proposed method (D-SCSformer) over the second-best method is statistically significant at the *p* < 0.05/*p* < 0.01 level, respectively.

**Table 6:** Ablation experiment table.

Model	MSE	MAE
SCSformer	0.30086862146638544	0.3685976541042328
Model 1	0.25244804322719574	0.3390711915490115
Model 2	0.28954741954803467	0.3556258857250136
D-SCSforme	**0.20124180614948273**	**0.2884479761123657**

Note: Bold values denote the optimal results, while underlined values represent the second-best results.

## Data Availability

The raw data supporting the conclusions of this article will be made available by the authors on request.

## Appendix A

**Table A1:** Summary of experimental design, replication scheme, and sample size justification.

Item	Specification
Storage treatments (temperature/RH)	15 °C/65% RH; 25 °C/70% RH; 35 °C/75% RH
Storage setup	Three constant temperature–humidity chambers (one chamber per treatment)
Biological replicates (per treatment)	*n* = three independent biological replicates
Sample amount (per biological replicate)	250 g shelled peanut kernels; sealed in separate breathable bags
Total independent storage units	Three treatments × three replicates = nine units
Sampling frequency and duration	Daily sampling for 210 days (30 weeks); each biological replicate sampled independently at each time point
Sample preservation	Immediately flash-frozen in liquid nitrogen and stored at −80 °C until analysis
Technical replicates (per indicator)	Triplicate technical replicates for each quality indicator
LC–MS repeat injections	Each extract injected twice
Environmental control and monitoring	Setpoints maintained by built-in chamber control (allowable fluctuation ±0.5 °C); T/RH automatically recorded at 10 min/1 h intervals
Modeling dataset (for time series prediction)	Daily record per condition (one record per “day × condition”); total 630 records (210 days × three conditions); chronological split 8:1:1 (train/val/test)
Power analysis (sample size justification)	Post hoc power analysis (α = 0.05; target power = 0.80) based on effect sizes estimated from the temperature/time factorial analysis of key indicators confirmed that the chosen replication (*n* = three biological replicates per condition with longitudinal repeated measurements) provided adequate statistical power (≥0.80) to detect treatment and interaction effects.

Each temperature–humidity treatment comprised three independent biological replicates (250 g per replicate) stored in dedicated constant temperature–humidity chambers, and samples were collected daily for 210 days, immediately flash-frozen, and stored at −80 °C until analysis. The quality indicators were quantified using triplicate technical replicates (LC–MS extracts injected twice), while chamber setpoints were maintained within ±0.5 °C and automatically logged (10 min/1 h); a post hoc power analysis (α = 0.05, target power = 0.80) based on observed effect sizes confirmed that the replication strategy provided adequate statistical power to detect treatment-related differences.

**Table A2:** Hyperparameter settings used in experiments.

Category	Hyperparameter	Symbol/Arg	Value (Used)	Notes/Source
Reproducibility	Random seed	fix_seed	2021	random/torch/numpy all set to 2021
Task	Task type	task_name	long_term_forecast	overridden after parse_args()
Data	Dataset type	data	custom	overridden
Data	Feature mode	features	M	multivariate→multivariate
Data	Target variable	target	b	mainly used for S/MS
Data	Time index keys	date_index	[‘SecondOfMinute’]	used for time feature stats/encoding
I/O dims	Encoder input dim	enc_in	6	overridden
I/O dims	Decoder input dim	dec_in	6	overridden
I/O dims	Output dim	c_out	6	overridden
Window/Horizon	Input length	seq_len	16	sliding window length (16 days/steps)
Window/Horizon	Label length	label_len	16	decoder start token length
Window/Horizon	Prediction length	pred_len	16	forecast horizon
Architecture	Model dimension	d_model	1024	overridden
Architecture	FFN dimension	d_ff	512	overridden
Architecture	Attention heads	n_heads	8	default (not overridden)
Architecture	Encoder layers	e_layers	2	overridden
Architecture	Decoder layers	d_layers	1	overridden
Architecture	Attention factor	factor	3	overridden
Architecture	Dropout	dropout	0.1	default (not overridden)
Architecture	Embedding type	embed	timeF	default
Architecture	Activation	activation	gelu	default
Architecture	Distillation	distil	True	default
Extra module	Origin coef	orgin_coef	1.0	overridden (Inception/FDNet-like)
Extra module	RevIN coef	revin_coef	0.0	overridden
Extra module	ConvD coef	convd_coef	1.0	overridden
Extra module	Conv kernel size	kernel_size	3	overridden
Training	Batch size	batch_size	32	default (not overridden)
Training	Max epochs	train_epochs	1000	overridden
Training	Early stop patience	patience	3	overridden
Optimization	Learning rate	learning_rate	0.001	overridden
Optimization	Loss	loss	MSE	default
Optimization	LR schedule	lradj	type5	overridden
Runs	Repeated runs	itr	1	overridden

**Table A3:** Abbreviations.

Abbreviation	Meaning
PV	Peroxide Value
AV	Acid Value
T/RH	Temperature/Relative Humidity
DCN	Deep Clustering Network
DEC	Deep Embedded Clustering
SC/DB/CH	Silhouette/Davies–Bouldin/Calinski–Harabasz

**Table A4:** Notation and dimensions.

Symbol	Meaning	Dimension
$x\in R^{T\times M}$	multivariate input time series	T: time length, M: variables
$x_{t}$	observation vector at time t	$R^{M}$
$y_{t+h}$	target at horizon h	$R^{M}$ or selected indicators
T	input sequence length	time steps (days)
H	prediction horizon	time steps
d	embedding dimension	scalar
s	segment length	time steps
W	model parameters	—
Q,K,V	query/key/value in attention	R′
